# Development and evaluation of a manual segmentation protocol for deep grey matter in multiple sclerosis: Towards accelerated semi-automated references

**DOI:** 10.1016/j.nicl.2021.102659

**Published:** 2021-04-06

**Authors:** Alexandra de Sitter, Jessica Burggraaff, Fabian Bartel, Miklos Palotai, Yaou Liu, Jorge Simoes, Serena Ruggieri, Katharina Schregel, Stefan Ropele, Maria A. Rocca, Claudio Gasperini, Antonio Gallo, Menno M. Schoonheim, Michael Amann, Marios Yiannakas, Deborah Pareto, Mike P. Wattjes, Jaume Sastre-Garriga, Ludwig Kappos, Massimo Filippi, Christian Enzinger, Jette Frederiksen, Bernard Uitdehaag, Charles R.G. Guttmann, Frederik Barkhof, Hugo Vrenken

**Affiliations:** a Radiology and Nuclear Medicine, MS Center Amsterdam, Amsterdam Neuroscience, Amsterdam UMC, Location VUmc, Amsterdam, NL, Netherlands; b Department of Neurology, MS Center Amsterdam, Amsterdam Neuroscience, Amsterdam UMC, Location VUmc, Amsterdam, NL, Netherlands; c Center for Neurological Imaging, Department of radiology, Brigham and Women’s Hospital, Harvard Medical School Boston, MA, USA; d Department of Human Neurosciences, “Sapienza” University of Rome, Rome, IT, Italy; e Department of Neurosciences, San Camillo Forlanini Hospital, Rome, IT, Italy; f Institute of Neuroradiology, University Medical Center Goettingen, Goettingen, DE, Germany; g Department of Neurology, Medical University of Graz, Graz, AT, Austria; h Neuroimaging Research Unit, Institute of Experimental Neurology, Division of Neuroscience, United States; i Neurology Unit, San Raffaele Scientific Institute, UniSR, Milan, IT, Italy; j Division of Neurology and 3T MRI Research Center, Department of Advanced Medical and Surgical Sciences, University of Campania “Luigi Vanvitelli”, Naples, IT, Italy; k Department of Anatomy and Neurosciences, MS Center Amsterdam, Amsterdam Neuroscience, Amsterdam UMC, Vrije Universiteit Amsterdam, Amsterdam, NL, Netherlands; l Medical Image Analysis Center (MIAC), United States; m Neurologic Clinic and Policlinic and Neuroradiology, Department of Biomedical Engineering, University Hospital Basel, Basel, CH, Switzerland; n Department of Neuroinflammation, Institute of Neurology, UCL, London, UK; o Section of Neuroradiology and MRI Unit, Department of Radiology, University Hospital Valld’Hebron, Autonomous University of Barcelona, Barcelona, ES, Spain; p Deptartment of Diagnostic and Interventional Neuroradiology, Hannover Medical School, Hannover, DE, Germany; q Department of Neurology, University Hospital iValld’Hebron, Autonomous University of Barcelona, Barcelona, ES, Spain; r Neurophysiology Unit, San Raffaele Scientific Institute, Italy; s Vita-Salute San Raffaele University, Milan, IT, Italy; t Division of Neuroradiology, Vascular and Interventional Radiology, Department of Radiology, Medical University of Graz, Graz, AT, Austria; u Department of Neurology, Glostrup University Hospital, Copenhagen, DK, Denmark; v Institutes of Neurology & Healthcare Engineering, UCL, London, UK

**Keywords:** Multiple Sclerosis, MRI, Deep grey matter, Atrophy, Segmentation, Reference set

## Abstract

**Background:**

Deep grey matter (dGM) structures, particularly the thalamus, are clinically relevant in multiple sclerosis (MS). However, segmentation of dGM in MS is challenging; labeled MS-specific reference sets are needed for objective evaluation and training of new methods.

**Objectives:**

This study aimed to (i) create a standardized protocol for manual delineations of dGM; (ii) evaluate the reliability of the protocol with multiple raters; and (iii) evaluate the accuracy of a fast-semi-automated segmentation approach (FASTSURF).

**Methods:**

A standardized manual segmentation protocol for caudate nucleus, putamen, and thalamus was created, and applied by three raters on multi-center 3D T1-weighted MRI scans of 23 MS patients and 12 controls. Intra- and inter-rater agreement was assessed through intra-class correlation coefficient (ICC); spatial overlap through Jaccard Index (JI) and generalized conformity index (CIgen). From sparse delineations, FASTSURF reconstructed full segmentations; accuracy was assessed both volumetrically and spatially.

**Results:**

All structures showed excellent agreement on expert manual outlines: intra-rater JI > 0.83; inter-rater ICC ≥ 0.76 and CIgen ≥ 0.74. FASTSURF reproduced manual references excellently, with ICC ≥ 0.97 and JI ≥ 0.92.

**Conclusions:**

The manual dGM segmentation protocol showed excellent reproducibility within and between raters. Moreover, combined with FASTSURF a reliable reference set of dGM segmentations can be produced with lower workload.

## 1 Introduction

Patients with multiple sclerosis (MS) exhibit damage of the grey matter (GM), including focal lesions and atrophy. (Du Toit et al., 2008, Bagnato et al., 2006, Geurts et al., 2005) GM atrophy can be quantified from structural brain magnetic resonance images (MRI) and has become an important and clinically relevant imaging outcome measure of MS. In particular, atrophy of deep GM (dGM) structures such as the caudate nucleus, putamen and thalamus has become of interest in MS, as it has been shown to correlate with important clinical outcome such as cognition. (Schoonheim et al., 2015, Bishop et al., 2017, Bermel et al., 2003, Houtchens et al., 2007, Pagani et al., 2005) Atrophy measures of the dGM may serve as potential imaging biomarkers in MS. However, the applicability for everyday clinical use is limited, in part because there is a so far unmet need for reliable automated segmentation methods. (Wattjes et al., 2015, Sastre-Garriga et al., 2020)

Current state-of-the-art and frequently used automated segmentation methods suffer from substantial limitations with respect to both reproducibility and accuracy, which is partly due to the presence of MS pathological changes. (Popescu et al., 2014, Popescu et al., 2016, Gelineau-Morel et al., 2012, Meijerman et al., 2018, Amiri et al., 2018, de Sitter et al., 2020) Specifically, there are various confounds that can affect the measurement of dGM atrophy: image registration and segmentation can be negatively affected by the presence of white matter lesions, (Gelineau-Morel et al., 2012, de Sitter et al., 2020) generalized or local atrophy, or subtle tissue contrast changes (Amiri et al., 2018, Westlye et al., 2009). To achieve accurate automated dGM segmentation in the presence of MS abnormalities, it is important that new methods are validated against expert reference outlines of dGM in representative MS samples. Therefore, we developed a standardized protocol for manually delineating the caudate, putamen and thalamus on 3D T1-weighted MRI and evaluated its quality in terms of reliability within and amongst multiple expert raters, using a multi-center MS imaging dataset.

To validate the automated methods for measuring dGM atrophy, a more complete analysis in a larger multi-center set of image volumes is required. Since manual outlining is difficult, labor-intensive and time-consuming, (Grimaud et al., 1996, Paty et al., 1986, Fischl et al., 2002) we endeavored to reduce the workload by reconstructing full semi-automated segmentations from sparse delineations as input. Specifically, we investigated the performance of a recently developed semi-automated technique called ‘FAst Segmentation Through SURface Fairing’ (FASTSURF), (Bartel et al., 2019) which was demonstrated as a proof-of-concept for the hippocampus in Alzheimer patients by Bartel et al. (2019). Since this technique exhibited excellent accuracy for hippocampus, we hypothesized that FASTSURF can also be used to generate accurate reference segmentations of various other brain structures, with substantially lower workload than full manual tracings. This may provide an important impetus towards improved segmentation of dGM. In future work, when this protocol is applied, such segmentations can be used to train or optimize automated methods such that these will segment the structures of interest well in MS cases.

To summarize, in this study we aimed first, to develop a standardized protocol for manually tracing the caudate, putamen and thalamus. Secondly, the reliability of the protocol was investigated with multiple expert readers, on multi-center MS images. Thirdly, we evaluated the accuracy of FASTSURF to reconstruct full segmentations of the dGM in which sparse delineations served as input.

## 2 Materials and methods

### 2.1 Dataset and MRI acquisition

Brain MRI scans of 12 healthy controls (HCs) (8 females) and 23 MS patients (12 females) from nine centers were retrospectively included, which were all acquired as part of two previously described MAGNIMS studies (www.magnims.eu). (Rocca et al., 2014, Ropele et al., 2014) The sample used for this study was selected to ensure that: many different MR scanners were included, most of the patients had progressive MS disease course types, and that the distributions of sex and age were closely matched to the overall dataset. The HCs were matched with the MS patients on scanner type, sex and age. Demographics of the subjects are shown in Table 1. Table 2 shows the number of subjects per center (MR scanner). All local institutional review boards approved the original study and written informed consent had been obtained from all participants. MR imaging was performed on 3.0 Tesla whole-body MR systems, and near-isotropic, ~1mm (Geurts et al., 2005) voxel size, 3D T1-weighted datasets were included. Details on image acquisition parameters used in each center are listed in Table 2.

**Table 1:** Demographics of healthy controls and MS patients.

Set	Type	N^a^	Age in years^b^	Disease types	DD in years^b^	EDSS^c^
Total	HC	12 (8)	38.4 ± 7.8			
	Patient	23 (12)	42.9 ± 9.9	11 RR, 5 SP, 7 PP	11.6 ± 6.9	2.5 (2.5)
Training	HC	5 (5)	34.7 ± 8.0			
	Patient	12 (6)	44.4 ± 11.9	7 RR, 2 SP, 3 PP	12.1 ± 8.3	2.0 (2.5)
Test	HC	7 (3)	41.1 ± 7.1			
	Patient	11 (6)	41.3 ± 7.4	4 RR, 3 SP, 4 PP	11.1 ± 5.59	3.5 (2.5)

^a^Number of subjects (Number of females).

^b^Mean ± standard deviation.

^c^Median (range).

Abbreviations: HC = healthy control, DD = disease duration, EDSS = expanded disability status scale, RR = relapsing-remitting, SP = secondary-progressive, PP = primary-progressive.

**Table 2:** An overview of the acquisition parameters for each center.

Institute	N^a^	Scanner manufacturer, scanner type	TR (ms)	TE (ms)	TI (ms)	FA (◦)	Acquisition (Voxel size (mm^3^)
A	13	GE, Signa HDxt	7.8	3	450	12	256x256x188 (0.976x0.976x1)
B	2	Siemens, Trio	2300	2.98	900	9	232x256x176 (1x1x1)
C	2	Siemens, Trio	1570	2.70	900	9	160x256x256 (1x1x1)
D	1	Philips, Achieva	6.9	2.78	831	9	160x240x240 (1x1x1)
E	5	Siemens, Trio	1900	2,1	900	9	224x256x176 (1x1x1)
F	2	Siemens, Trio	2200	2,94	900	10	256x192x192 (1x1x1)
G	5	Philips, Achieva	8,3	3,72	1000	8	256x256x192 (1x1x1)
H	2	GE, Signa HDxt	5,5	1,76	450	10	256x256x188 (1x1x1)
I	1	Philips, Achieva	8,3	3,72	1000	8	256x256x192 (1x1x1)

^a^Number of subjects per institute. Abbreviations: TR = repetition time, TE = echo time, TI = inversion time, FA = flip angle.

### 2.2 Manual segmentation protocol

The segmentation protocol (see Supplementary File S1 for the full protocol) was specifically developed for manually tracing dGM structures on 3D T1-weighted MRI scans of MS patients, by neurologists and neuroradiologists with broad experience in the field of MS and MRI, supervised by neuroradiologists (F.B. with>30 years of experience and M.P.W. with>20 years of experience). Together, we reviewed the literature and studied images of histopathological specimens, MRI, (stereotactic) anatomy and computational 3D reconstructions; most of which are also listed in the protocol as recommended study material for the readers, since they help to understand the 3D anatomical position/location and shape of the structure of interest in the human brain. Anatomical definitions were specified for each structure, supported in the protocol with example images from our own dataset. Alongside the anatomical landmarks, strict guidelines on how to recognize the outermost edges of the structures on orthogonal planes were described. Certain decisions on whether to include the geniculate bodies as part of the thalamus and how to distinguish the caudate and the putamen from the nucleus accumbens were based on a mixture of literature studies, expert opinion and practical reasoning.

Practically, the segmentation procedure consisted of two phases. First, demarcating the edges of the dGM structures on orthogonal slices, and second, tracing and fill the inside of the path defined by the reader in the axial plane, respecting the boundaries previously defined and the anatomical definitions that were specified for each structure.

### 2.3 Manual tracing

Manual outlining was performed within the online framework of the SPINE virtual laboratory (https://spinevirtuallab.org/), developed by the Center for Neurological Imaging (CNI) at Brigham and Women’s Hospital. This web-based program allows visualization of MR images in axial, coronal, and sagittal orientations to facilitate 3D anatomical interpretation. The voxel-wise labeling process was completely manual. It involved no thresholding, seed-growing, shape fitting or other automated interference. Following the segmentation protocol described above and presented in Supplementary File S1, three expert readers manually delineated the caudate nucleus, putamen and thalamus as a whole on axial slices, in a slice-by-slice manner, for all 35 images. The readers were a trained neurologist (J.B.), neuroscientist (J.S.) and neurologist (S.R.), blinded to the subject characteristics. To assess the intra-rater variability, a random subset of dGM structures for 3 subjects (1 HC and 2 MS patient) were delineated a second time by all 3 raters in a separate session more than three months later. To assess the validity of FASTSURF, another subset of six subjects (2 HC and 4 MS patients) were delineated in a separate session by one reader (J.B.), which included only 10 predefined axial slices per structure.

### 2.4 Reconstructions from sparse delineations: FASTSURF method

To allow construction of reference segmentations with reduced workload, the possibility of reconstructing full segmentations from sparse delineations was investigated. For this purpose, the semi-automated segmentation method FASTSURF was used, which is based on mesh processing procedures using a surface fairing technique that has been described in detail previously. (Bartel et al., 2019) Briefly, to reduce the delineation time for manual observers, only a few contours have to be outlined, at regular slice intervals. First, these sparse contours are interpolated so that each contour has the same number of points. A closed mesh is then constructed by placing intermediate contours between the known contours. Vertex positions for the intermediate contours are obtained by solving the following bi-Laplacian system of equations for the unknown *x*, *y* and *z*-coordinates of the vertices of the intermediate contours:

$$\sum_{m}{L_{n,m}}^{2}x_{m}=\sum_{m}{L_{n,m}}^{2}y_{m}=\sum_{m}{L_{n,m}}^{2}z_{m}=0$$

in which the Laplacian filter *L_n,m_* represents the connectivity graph with *n* and *m* being the mesh vertices. Solving these equations leads to a smooth surface mesh passing through the delineated points with minimum curvature.

Originally FASTSURF was designed for the hippocampus. In the present study, we quantitatively investigated this application for segmentation of the thalamus, caudate nucleus and putamen. First, similar to the approach of Bartel et al. (2019), sparse contours were extracted from fully manually segmented structures. The segmented structures were converted to meshes using the marching cubes algorithm and sparse contours were extracted at regular intervals, which served as input for FASTSURF (From now on: ‘FASTSURF with sparse contours’).

Second, in a small subset of six images, one of the raters manually traced 10 predefined contours for each structure *de novo*, i.e., without creating outlines of the structures on the intermediate slices. These 10 *de novo* contours were used as input for FASTSURF (From now on: ‘FASTSURF with *de novo* contours’). This allowed us to evaluate whether the protocol can be combined with FASTSURF to reconstruct full segmentations of the dGM using only 10 *de novo* delineations as input.

Extra information on sparse contour simulation and training of FASTSURF can be found in the supplementary data (File S2 and Table S1-S4).

### 2.5 Quantitative performance analysis

**Fig. 1:**
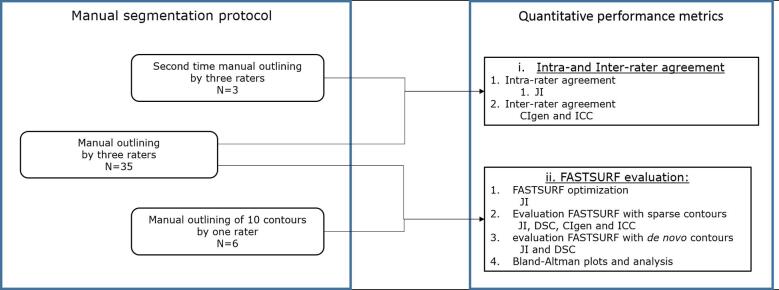
**Overview of study design.** A flowchart of the study design divided in two boxes; 1) manual segmentation protocol and 2) quantitative performance metrics. The manual segmentation protocol was used to create three datasets; quantitative performance metrics were used to assess spatial (JI, DSC and CIgen) and volumetric agreement (ICC). Abbreviations: CIgen = generalized conformity index; ICC = intraclass correlations; DSC = Dice Similarity Coefficient, JI = Jaccard index; N = number of subjects.

In Fig. 1 an overview of the study design is shown. The two main quantitative performance metrics are; (i) intra- and inter-rater agreement of manual outlines of 3 raters on 35 images (ii) accuracy of FASTSURF in terms of volumetric and spatial agreement. In the next subparagraphs more details are described on the experiment and the statistical analyses.

### 2.6 Intra- and inter-rater agreement of manual tracings

The manual outlines of the three raters were evaluated on both intra-rater and inter-rater reliability.

Intra-rater agreement was assessed spatially with the Jaccard Index (JI); $\mathrm{JI}=\frac{V_{i\cap j}}{V_{i\cup j}}$ between the first and second manual tracing of the structures. $V_{i\cap j}$ is volume of intersection of outline *i* and *j* and $V_{i\cup j}$ is volume of union of outline *i* and *j*.
Inter-rater spatial agreement was assessed spatially with the generalized conformity index (CIgen), (Kouwenhoven et al., 2009) which is essentially a generalization of the Jaccard index for multiple raters; a full definition and explanation is provided in the supplementary File S3. Volumetrically, a two-way mixed effects model for intraclass correlation coefficients (ICC) using an absolute agreement definition was measured between the three raters (Shrout and Joseph, 1979).

### 2.7 Fastsurf

The performance of FASTSURF for dGM structures was evaluated in four ways; a) optimization of FASTSURF parameters; b) optimized FASTSURF with sparse delineations from full segmentations as input; c) optimized FASTSURF with 10 *de novo* contours as input; and d) agreement between the expert manual labels and ‘FASTSURF with sparse contours’ and ‘FASTSURF with *de novo* contours’ using Bland-Altman plots.

For optimization of FASTSURF parameters, the dataset was divided into a training set (N = 17) and a test set (N = 18). In both groups, the different centers and numbers of patients and controls were equally distributed (see Table 2). The training set was used to find the optimal settings for the parameters of FASTSURF and the test set was used to study the performance of optimized FASTSURF compared to the manual outlines.
The optimal settings obtained from the training set were applied in the test sets of each rater’s segmentations separately. Optimal settings can be found in Supplementary Table 5; for contours the optimal setting was 10. The spatial agreement between the resulting three datasets of ‘FASTSURF with sparse contours’ were evaluated with CIgen. The results were compared to the inter-rater agreement of the expert manual outlines.

Additionally, the segmentations of all 3 raters were pooled as one dataset, which served to compare ‘FASTSURF with sparse contours’ to the manual references on both volumetric as spatial agreement. Volumetrically, the agreement was quantified with the ICC for absolute agreement; (Koch, 1982) spatial agreement was assessed through the JI and Dice Similarity Coefficient (DSC) between ‘FASTSURF with sparse contours’ and manual references. With DSC = 2TP/(2TP + FP + FN), with TP, FP and FN, respectively True Positive, False Positive and False Negative.

‘FASTSURF with *de novo* contours’ was validated on six images containing only 10 contours of each structure as input by one rater (J.B.). The segmentations that were obtained through ‘FASTSURF with *de novo* contours’ were compared to the manual reference on spatially agreement (JI and DSC), and compared with the agreement between ‘FASTSURF with sparse contours’ and manual reference.
To evaluate the agreement between the fused manual labels and ‘FASTSURF with sparse contours’ and ‘FASTSURF with *de novo* contours’, Bland-Altman plots were created in which the difference of two paired measurements [(A-B)] was plotted against the average of the two measurements [(A + B)/2], (Giavarina, 2019, Altman, 1983) with separate colors for MS and controls to visually inspect whether there are disease specific effects. We ran a paired sample *t*-test (two-sided) to examine whether the mean of the difference equals 0.

### 2.8 Interpretation of statistical results

JI, DSC and CIgen range between 0 and 1, where perfect overlap yields a JI, DSC or CIgen value of 1, and no overlap yields a JI, DSC or CIgen value of 0. A JI or CIgen > 0.7 and a DSC > 0.8 is regarded as excellent. (Bartko, 1991)

ICC also ranges between 0 and 1. We used Altman’s criteria to interpret the ICCs: <0.40 was considered as poor reliability, 0.40 to 0.74 was considered fair to good, and ≥ 0.75 was considered excellent. (Cicchetti, 1994)

## 3 Results

**Fig. 2:**
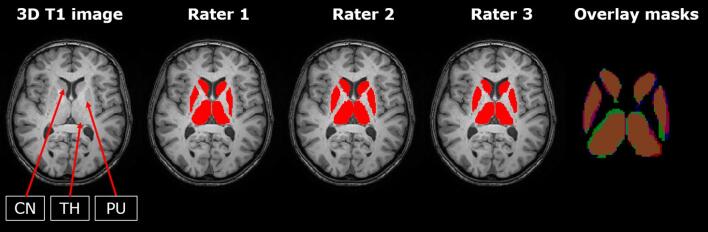
**Overview of manual delineations of the 3 raters and their overlap.** From left to right: Axial 3D T1-weighted MRI slice with segmentations, 2D view of manual reference of rater 1 to 3 and 2D view of overlap of raters with green, blue and red one rater, purple and pink two and orange three raters. (For interpretation of the references to colour in this figure legend, the reader is referred to the web version of this article.) Abbreviations: CN = caudate nucleus; TH = thalamus; PU = putamen.

**Fig. 3:**
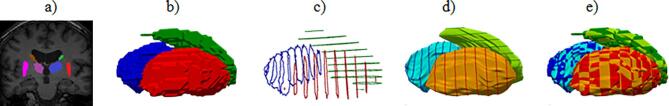
**Overview of manual delineation of one rater and FASTSURF segmentations. (**a) the tracings of one rater; (b) the extracted contours from full segmentations which were used for ‘FASTSURF with sparse contours’; (c) the reconstructed FASTSURF segmentations for the caudate, putamen and thalamus; (d) an intersection of ‘FASTSURF with sparse contours’ and the manual references in 3D; and (e) the overlap of c and d.

Fig. 2 shows example images of dGM delineations for each rater separately and their overlap. Fig. 3 shows the tracings of one rater and the reconstructed FASTSURF segmentations for the caudate, putamen and thalamus.

### 3.1 Intra- and inter-rater agreement of manual tracings

The intra-rater agreement on spatial overlap was excellent with a mean (across raters) JI of 0.83 ± 0.11, 0.86 ± 0.05 and 0.86 ± 0.10 for the caudate nucleus, putamen and thalamus, respectively.

The inter-rater agreement on spatial overlap was excellent; with both left and right hemisphere pooled together, the average CIgen for the caudate nucleus, putamen and thalamus were 0.74 ± 0.05, 0.74 ± 0.06 and 0.75 ± 0.06 respectively. The volumetric agreement between the raters was also excellent with an ICC of 0.76 for the caudate, 0.79 for the putamen and 0.79 for the thalamus (left and right hemisphere pooled together). Table 3 provides average CIgen and ICC values for all structures, both for each hemisphere separately and averaged.

**Table 3:** Inter-rater agreement between the three raters; the generalized conformity index (CIgen) and intra-class correlations (ICC) between raters, separated for structure and hemisphere.

Structure	Hemipshere	CIgen^a^	ICC
Caudate	Both	0.738 ± 0.049	0.762
	Left	0.733 ± 0.054	0.771
	Right	0.753 ± 0.042	0.766
Putamen	Both	0.736 ± 0.059	0.794
	Left	0.728 ± 0.061	0.769
	Right	0.753 ± 0.049	0.833
Thalamus	Both	0.746 ± 0.058	0.785
	Left	0.762 ± 0.039	0.815
	Right	0.741 ± 0.072	0.762

Abbreviations: CIgen = generalized conformity index, ICC = intra-class correlations.

a Mean ± standard deviation.

### 3.2 Fastsurf

#### 3.2.1 Parameter optimization for FASTSURF

The results of the parameter optimization for the FASTSURF software, carried out on the test set (N = 17) are shown in the supplementary Tables S1-S5. The parameters that were optimized were: the orientation of outlining planes, the number of the outlined contours, the number of intermediate contours added by FASTSURF between two outlined contours and the number of points used for each contour.

#### 3.2.2 Agreement of ‘FASTSURF with sparse contours’

In the test set (N = 18), the performance of optimized FASTSURF was quantitatively evaluated. In Table S6 the CIgen values for ‘FASTSURF with sparse contours’ are provided for all structures bilaterally, as well as averaged across hemispheres. Inter-rater agreement on spatial overlap for ‘FASTSURF with sparse contours’ was almost identical to the agreement between expert manual references.

The volumetric and spatial agreement of ‘FASTSURF with sparse contours’ with manual reference segmentations was excellent (Table 4), with total bilateral volume ICCs for absolute agreement of 0.979 for the caudate nucleus, 0.999 for the putamen and 0.999 for the thalamus and mean JI of 0.92 ± 0.02, 0.95 ± 0.01, 0.96 ± 0.02, respectively.

**Table 4:** ICC, Jaccard Index and Dice Similarity Coefficient between manual references and ‘FASTSURF with sparse contours’ and manuala references.

Structure	Hemispheres	ICC	Jaccard Index^a^	Dice Similarity Coefficient ^a^
Caudate	Both	0.979	0.918 ± 0.023	0.924 ± 0.026
	Left	0.984	0.920 ± 0.021	0.925 ± 0.028
	Right	0.973	0.914 ± 0.024	0.923 ± 0.025
Putamen	Both	0.999	0.952 ± 0.013	0.960 ± 0.019
	Left	0.999	0.951 ± 0.012	0.958 ± 0.020
	Right	0.999	0.954 ± 0.013	0.961 ± 0.017
Thalamus	Both	0.999	0.962 ± 0.021	0.967 ± 0.030
	Left	0.999	0.960 ± 0.023	0.965 ± 0.030
	Right	0.999	0.964 ± 0.019	0.970 ± 0.030

^a^Mean ± standard deviation.

Abbreviations: ICC = the intraclass correlation coefficient (two-way mixed model with absolute agreement).

#### 3.2.3 Agreement of ‘FASTSURF with *de novo* contours’

The average volumes of the reconstructed dGM using ‘FASTSURF with *de novo* contours’ are displayed in Table 5, alongside the average volumes of the manual reference tracings and segmentations from ‘FASTSURF with sparse contours’ for the same six subjects. Furthermore, the JI between FASTSURF results and the manual references are shown. The average JI between ‘FASTSURF with *de novo* contours’ and the manual segmentations were in the same range as the overlap between ‘FASTSURF with sparse contours’ and the manual references.

**Table 5:** Average volumes (mL) of manual reference, FASTSURF with sparse contours and FASTSURF with *de novo* contours. And the average Jaccard Index (JI) and Dice Similarity Coefficient (DSC) between manual reference and FASTSURF with sparse contours and with *de novo* contours. ^a.^

N = 6		Manual	FASTSURF with sparse contours			FASTSURF with *de novo* contours		
Structure	Hemispheres	Volume	Volume	Jaccard Index	Dice Similarity Coefficient	Volume	Jaccard Index	Dice Similarity Coefficient
Caudate	Both	4.64 ± 0.68	4.39 ± 0.51	0.823 ± 0.042	0.900 ± 0.011	4.38 ± 0.64	0.798 ± 0.042	0.923 ± 0.030
	Left	4.66 ± 0.73	4.45 ± 0.55	0.822 ± 0.045	0.901 ± 0.011	4.43 ± 0.75	0.797 ± 0.043	0.923 ± 0.035
	Right	4.62 ± 0.69	4.34 ± 0.52	0.823 ± 0.042	0.900 ± 0.011	4.33 ± 0.58	0.800 ± 0.041	0.922 ± 0.027
Putamen	Both	5.44 ± 1.09	5.38 ± 1.01	0.884 ± 0.035	0.943 ± 0.008	5.41 ± 1.07	0.880 ± 0.039	0.947 ± 0.022
	Left	5.54 ± 1.23	5.42 ± 1.08	0.883 ± 0.042	0.944 ± 0.009	5.36 ± 0.81	0.883 ± 0.040	0.944 ± 0.030
	Right	5.35 ± 1.05	5.33 ± 1.02	0.886 ± 0.030	0.943 ± 0.007	5.46 ± 1.17	0.877 ± 0.038	0.950 ± 0.022
Thalamus	Both	6.83 ± 0.83	6.70 ± 0.79	0.893 ± 0.032	0.938 ± 0.015	6.74 ± 0.79	0.887 ± 0.035	0.953 ± 0.35
	Left	6.83 ± 0.84	6.75 ± 0.80	0.885 ± 0.037	0.927 ± 0.012	6.78 ± 0.77	0.877 ± 0.032	0.948 ± 0.037
	Right	6.83 ± 0.90	6.64 ± 0.85	0.892 ± 0.033	0.947 ± 0.012	6.69 ± 088	0.901 ± 0.036	0.958 ± 0.035

^a^ mean ± standard deviation.

Abbreviations: mL = milliliter, N = number of subjects.

#### 3.2.4 Bland-Altman plots and analysis

**Fig. 4:**
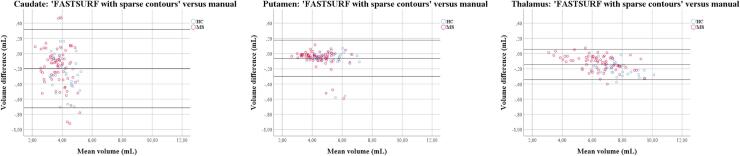
**Bland Altman scatter plots of the deep grey matter volume measurements of the MS patients and controls for ‘FASTSURF with sparse contours’.** The difference of two paired measurements [(FASTSURF–manual)/average] was plotted against the average of the two measurements [(FASTSURF + manual)/2].

Fig. 4, and Table 6 show the results of the Bland-Altman scatter plots and analysis of the combined (left + right) dGM volume measurements: FASTSURF *minus* the combined expert manual labels; with separate labels for MS patients and controls. For all structures, FASTSURF obtained smaller volumes (mL) than the manual output [mean difference (SD): caudate: −0.20 (0.26); putamen: −0.06 (0.12); thalamus: −0.14 (0.10), all *p-values* < 0.001]. Visual inspection of the data revealed the same effects in the MS patients and controls. Because of the small number of subjects (N = 6) in ‘FASTSURF with the novo contours’ we were unable to perform similar analysis (For scatter plot see supplementary figure 1).

**Table 6:** Pairwise Bland-Altman comparisons between ‘FASTSURF with sparse contours’ and combined expert manual labels.

Structure	Subjects	µ diff	SD	SE µ	p-Value
Caudate	Total	−0.20	0.26	0.03	<0.001
	HC	−0.22	0.25	0.04	<0.001
	Patients	−0.19	0.26	0.03	<0.001
Putamen	Total	−0.06	0.12	0.01	<0.001
	HC	−0.07	0.13	0.02	0.003
	Patients	−0.06	0.11	0.01	<0.001
Thalamus	Total	−0.14	0.10	0.01	<0.001
	HC	−0.19	0.09	0.01	<0.001
	Patients	−0.12	0.10	0.01	<0.001

Abbreviations: HC = healthy controls; µ diff = mean difference; SD = standard deviation; SE µ= standard error of µ; p-value in bold represent significant values.

## 4 Discussion

In this study we presented a novel protocol with stringent guidelines for manually tracing the caudate nucleus, putamen and thalamus on 3D T1-weighted MR images, which exhibited excellent reliability in a multi-center dataset of MS patients. Moreover, we provided evidence that FASTSURF can be used to generate equally accurate dGM reference segmentations as high quality manual tracings of experienced raters.

The high levels of agreement between the experts’ manual outlines of the dGM structures (JI ~ 0.75, ICC ~ 0.78) indicate that our segmentation protocol can be used to create dGM reference datasets with sufficient levels of accuracy, even in multi-center settings. (Cicchetti, 1994, Bocchetta et al., 2015) Also, our data revealed that the described method can be used on conventional as well as more advanced 3D T1 images. In addition, this study demonstrated that FASTSURF can be used to generate accurate dGM measurements with *de novo* partial contours as input. This will ultimately reduce the workload and timely effort to create sufficient reference datasets for training and validation purposes of algorithms for measuring dGM atrophy in MS.

The output obtained through ‘FASTSURF with sparse contours’ as well as the segmentations from ‘FASTSURF with *de novo* contours’ showed high levels of agreement with the manual references, both volumetrically and spatially, indicating that semi-automation will not compromise the quality of the data. However, the Bland-Altman plots revealed that overall the volumes of FASTSURF were slightly lower than the manual annotations. This probably resulted from the location of the 10 predefined contours, which were distributed equally over the width of the structures. Therefore it could be that the widest part of the structure was not taken into account. Nevertheless, future studies should help to improve FASTSURF to ensure greater accuracy.

Visually, the Bland-Altman plots did not reveal clear disease specific effects on the agreement in MS patient versus controls. In future studies, more images with *de novo* partial contours obtained by multiple raters should be generated to further validate the accuracy of FASTSURF in this manner. Lastly, while FASTSURF was originally developed for the hippocampus in Alzheimer’s patients, (Bartel et al., 2019) our results conclusively demonstrated that this method is also accurate for cross-sectional segmentation of the dGM in MS patients. Future studies should investigate whether this technique is suitable for longitudinal observations as well.

Although the dGM manual segmentation protocol showed good reproducibility within and among raters, certain guidelines might be debated. Considering the thalamus, it was decided that the geniculate bodies should be included, as they form part of extensions of the structure itself. Hence, the thalamus comprises mixed WM-GM voxel intensities, which makes it rather difficult to separate different thalamic subparts from the background, especially in the presence of atrophy. (Houtchens et al., 2007, Fischl et al., 2002, Derakhshan et al., 2010) Therefore, in order to minimize error and reduce variability, we decided to delineate all dGM structures as a whole. Although for the thalamus it is clear that specific nuclei are more sensitive to the MS disease process, which was a limitation of this study. Furthermore, the nucleus accumbens is difficult to distinguish from adjacent structures due to close proximity to the caudate nucleus and putamen. Therefore, we used well-defined anatomical landmarks to identify the anterior and posterior limits of the nucleus accumbens in the coronal plane, and the bottom of the lateral ventricles as the inferior border of the caudate. (Lucas-Neto et al., 2013)

Interestingly, our data revealed slightly worse estimations of the caudate nucleus compared to the putamen and thalamus, both manually as well as with FASTSURF. This finding probably results from the different shapes of the structures. The tail of the caudate is substantially more elongated and curved compared to the other structures, and therefore difficult to trace unambiguously. Most importantly, our consistent approach allowed the readers to reproduce references with great accuracy. Incorporating features from advanced imaging techniques such as diffusion tensor imaging (DTI) or quantitative susceptibility mapping (QSM) would probably lead to more refined estimations of these boundaries. (Glaister et al., 2017, Daniel, 2017) However, the guidelines presented here were strictly based on 3D T1-weighted MRI, considering that this is the standard imaging contrast in clinical practice for these purposes. While this work focused on standard 3D T1-weighted imaging sequences that are readily available in a clinical setting, there have also been developments on other MR imaging techniques, such as MPRAGE with additional suppression of WM or GM, or susceptibility-based contrasts. (Tanner et al., 2012, Kecskemeti et al., 2016) While those techniques may not be ready yet for widespread clinical application, they could inform expert raters on the boundaries of the dGM structures, which could help training of improved automated methods, regardless of whether they are applied with or without direct input from these methods.

A possible limitation of this study was that we did not compare FASTSURF with other existing automated segmentation techniques However, two other studies that were recently published by our group already evaluated existing automated segmentations methods against manual references, using (partly) the same dataset (de Sitter et al., 2020, Burggraaff et al., 2020). Moreover, since this comparison would reveal any systematic difference between methods, e.g. with respect to anatomical definitions of the structures of interest, we argued that this would not be relevant for the value of creating accurate reference segmentations. Therefore, to maintain the focus of present work, we did not perform such statistical analysis. Another limitation of our study was that no statistical analysis was performed between ‘FASTSURF with de novo contours’ and manual annotations due to the limited sample size (N = 6). In future work, more manual delineations from trained expert raters should be included.

To conclude, we suggest that high-quality dGM segmentations can be created based on the proposed manual delineation protocol. Together with the standardized manual delineation protocol, FASTSURF can serve as an adequate tool to create accurate reference segmentations with considerably less effort than full manual outlines. This opens up possibilities for improving, training and developing algorithms for measuring dGM atrophy in MS and other neurodegenerative diseases.

## CRediT authorship contribution statement

**Alexandra Sitter:** Conceptualization, Formal analysis, Investigation, Methodology, Software, Visualization, Writing - original draft. **Jessica Burggraaff:** Conceptualization, Formal analysis, Investigation, Methodology, Visualization, Writing - original draft. **Fabian Bartel:** Formal analysis, Investigation, Software, Visualization, Writing - original draft, Writing - review & editing. **Miklos Palotai:** Investigation, Visualization. **Yaou Liu:** Investigation, Writing - review & editing. **Jorge Simoes:** Investigation, Writing - review & editing. **Serena Ruggieri:** Investigation, Writing - review & editing. **Katharina Schregel:** Investigation, Writing - review & editing. **Stefan Ropele:** Conceptualization, Investigation, Writing - review & editing. **Maria A. Rocca:** Conceptualization, Investigation, Writing - review & editing. **Claudio Gasperini:** . **Antonio Gallo:** Conceptualization, Investigation, Writing - review & editing. **Menno M. Schoonheim:** Conceptualization, Investigation, Writing - review & editing. **Michael Amann:** Conceptualization, Investigation, Writing - review & editing. **Marios Yiannakas:** Conceptualization, Investigation, Writing - review & editing. **Deborah Pareto:** Conceptualization, Investigation, Writing - review & editing. **Mike P. Wattjes:** Conceptualization, Investigation, Writing - review & editing. **Jaume Sastre-Garriga:** Conceptualization, Investigation, Writing - review & editing. **Ludwig Kappos:** Conceptualization, Investigation, Writing - review & editing. **Massimo Filippi:** Conceptualization, Investigation, Writing - review & editing. **Christian Enzinger:** Conceptualization, Investigation, Writing - review & editing. **Jette Frederiksen:** Conceptualization, Investigation, Writing - review & editing. **Bernard Uitdehaag:** Conceptualization, Investigation, Supervision, Writing - review & editing. **Charles R.G. Guttmann:** Conceptualization, Methodology, Formal analysis, Investigation, Supervision, Visualization, Writing - original draft, Writing - review & editing. **Frederik Barkhof:** Conceptualization, Methodology, Formal analysis, Investigation, Supervision, Visualization, Writing - original draft, Writing - review & editing. **Hugo Vrenken:** Conceptualization, Methodology, Formal analysis, Investigation, Supervision, Visualization, Writing - original draft, Writing - review & editing.

## Declaration of Competing Interest

The authors declare that they have no known competing financial interests or personal relationships that could have appeared to influence the work reported in this paper.
